# Evaluation of Functional Outcome in Surgically Managed Tibial Pilon Fractures

**DOI:** 10.7759/cureus.63242

**Published:** 2024-06-26

**Authors:** Raja Ramesh Badavath, Parineetha Akkala, Sundeep Kund Reddy Aluka, Chandrasekhar Patnala

**Affiliations:** 1 Orthopaedics, Nizam's Institute Of Medical Sciences, Hyderabad, IND

**Keywords:** distal tibia fractures, pilon fractures, foot and ankle, plafond, tibial pilon

## Abstract

Background

Pilon fractures are infrequent and among the most challenging to manage. One reason is the extensive soft tissue injury surrounding the distal tibia. Second, the articular surface of the distal tibia with a complex fracture pattern needs anatomic reduction. These fractures occur due to high energy impaction of the talus into the distal tibia. The fracture patterns and extent of soft tissue involvement vary based on the intensity of the impact's energy. The management needs to be patient-specific to prevent complications. Proper pre-operative planning with the help of computer tomography scans aids in choosing the approach and proper reduction. Either single-stage early definitive fixation or two-staged protocols involving the application of spanning external fixation to maintain length and allow soft tissue healing followed by definitive open reduction and internal fixation is done. However, complications still remain inevitable in a significant subset of patients.

Objective

To evaluate the functional outcome in surgically managed tibial pilon fractures using the American Orthopaedic Foot and Ankle Society (AOFAS) scoring system.

Methods

This prospective observational study included 20 patients who underwent surgery for pilon fractures of the tibia at Nizam's Institute of Medical Sciences between November 2020 and September 2022. The patients were between 18 and 65 years old and consented to participate in the study group. After undergoing patient-specific surgical management, all patients are followed for a minimum of six months. Their functional outcome is evaluated after fracture union and scheduled physiotherapy sessions every four weeks using the AOFAS scoring system. Ankle range of motion (ROM) is also evaluated.

Results

The average age of the patients was 40 years, and male predominance was present. Most of the patients (60%) underwent internal fixation. According to the AOFAS scoring system, six patients had an excellent outcome, 11 had a good outcome, and three had a fair outcome. Most of the patients (11 patients) had excellent to good ankle ROM. Complications were encountered in two patients with ankle stiffness and one with wound dehiscence.

Conclusion

Pilon fractures are more common in young adults due to road traffic accidents. The most common type of pilon fracture is a closed fracture, which can be treated with definitive internal fixation after the soft tissue has healed. Definitive internal fixation has shown excellent and good functional outcomes (according to the AOFAS score) with improved ankle ROM and no complications when compared to external fixation, which can result in ankle stiffness and delayed union.

## Introduction

Fractures occurring in the distal tibia, particularly those involving its articular surface, are identified as pilon fractures [1]. The term 'pilon' is often used interchangeably with the term plafond. In French, 'plafond' translates to 'ceiling,' reflecting the distal tibial articular surface's resemblance to the ceiling of the ankle joint. Treating distal tibial fractures poses significant challenges, primarily due to accompanying soft tissue injury [2]. The impact leads to the development of edema and fracture blisters. The complexity of the fracture pattern in the articular surface needs anatomical reduction. Metaphyseal bone loss in some patients requires bone grafting to maintain length and alignment. Surgical management has advanced considerably in recent years, primarily attributed to a better understanding of the importance of soft tissue and better radiological identification of fracture patterns, leading to better planning and improved implant designs. The surgical approach required is chosen based on the fracture pattern. These fractures are relatively rare and account for up to 5-7% of all tibia fractures [3]. These fractures typically occur due to axial loading, wherein the talus is forced into the distal tibia articular surface, leading to articular impaction. The positioning of the foot during impact, combined with the direction and intensity of the force, contributes to diverse fracture patterns and levels of comminution.

There are two classification systems that are commonly used, one being the Reudi and Allgower classification system [4]; it divides the fracture pattern into three types (type I, type II, and type III) based on the degree of comminution of the articular surface. The second one is the AO/OTA classification system [5]. This system categorizes the distal tibial fractures into three types: A, B, and C, which correspond to extra-articular, partial articular, and intraarticular, respectively, and sub-divided based on the degree of comminution. Type B3, C1, C2 and C3 are considered tibial pilon fractures.

Regardless of the classification system used, there exists a notable degree of variability between individual fracture patterns. A CT scan before surgery helps to better comprehend the fracture and plan surgical approaches and fixation techniques. Classically, 'span-scan-plan' is the protocol followed. Where an external fixator is placed across the ankle joint to facilitate soft tissue healing, followed by a CT scan to aid in precise surgical planning [6]. Open reduction and internal fixation with a plate is the most common surgical modality opted for.

The lower limb at this level has limited muscle coverage between the skin and bone, directly transferring impact energy to these soft tissue structures. The thin, soft tissue envelope surrounding the distal tibia, especially the subcutaneous nature of the bone on the medial aspect, poses challenges in fracture treatment.

With the growing occurrence of high-energy injuries, an increase in complications has been found, such as soft tissue dehiscence due to tension and inadequate soft tissue coverage, avascular necrosis, infection, osteomyelitis, delayed union, or non-union. Excessive dissection leading to altered blood supply in the region can lead to delayed healing and union.

The surgical plan must be formulated early at the initial presentation to determine whether single or staged procedures are required to achieve definitive fracture reduction. The ultimate goal of the management is to achieve union with minimum soft tissue injury and complications. The current study aims to evaluate the functional outcome in patients with pilon fractures and assess how the extent of soft tissue injury affects the functional outcome. It also discusses the complications of post-surgical management of pilon fractures.

## Materials and methods

After Institutional Ethics Committee approval, adult patients with tibial pilon fractures admitted to Nizam's Institute of Medical Sciences, Hyderabad, from March 2019 to February 2022, were taken into this study after attaining their informed, valid written consent. Clinical and radiological investigations necessary were carried out. Patients underwent surgical techniques based on their skin condition and fracture patterns. The following parameters are analyzed: age, gender, mode of injury, open or closed fracture, type of fixation (external or internal), ankle range of motion (ROM), and functional outcome using the American Orthopaedic Foot and Ankle Society (AOFAS) scoring system. 

**Figure 1:**
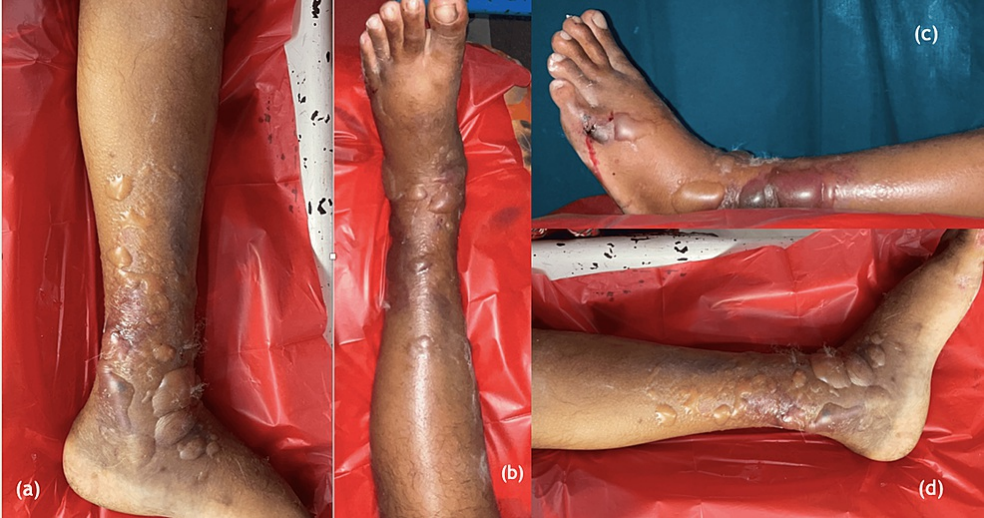
Fracture blisters post sustaining pilon fracture (a)&(d) on the medial aspect of the ankle and leg, (b) on anterior aspect of the ankle and leg, (c) on the lateral aspect

Patients between 18-65 years of age who sustained tibial pilon fractures, both open and closed fractures, were included in the study. Patients below the age of 18 years and above 65 years who are unfit for surgery or not willing to undergo surgery are excluded from the study. Data was collected from admission until discharge and outpatient follow-up for a minimum of six months. Patients with good skin condition were operated within a period of two to five days. Patients who developed blisters (Figure 1) due to very high energy impact were managed with the staged protocol of initial external fixation for the soft tissue to heal and then underwent definitive fixation. This method requires a second surgery and is a financial burden in certain government-driven health schemes. All cases were managed with a period of foot end elevation and medication until the skin condition improves for definitive fixation.

**Figure 2:**
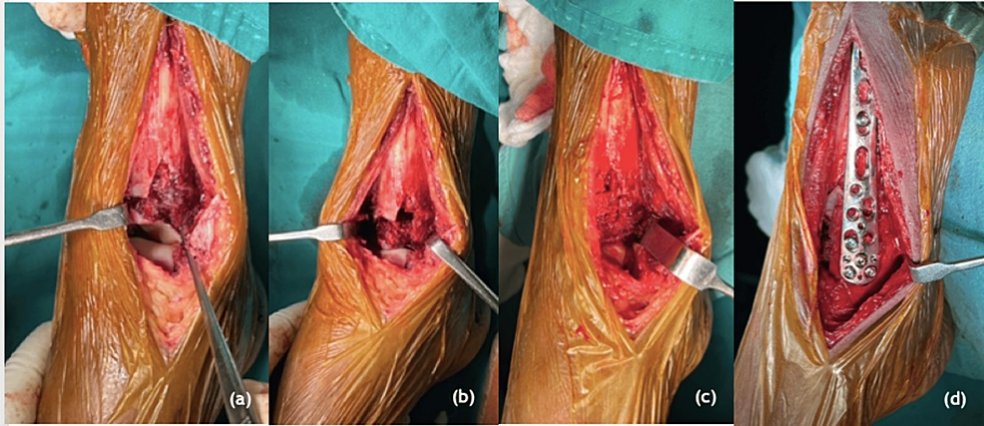
Bone grafting in tibial pilon fracture management (a) impaction the articular surface, (b) void after dis impaction, (c) void filled with iliac crest bone graft, (d) fixed with medial distal tibial plate

A few patients with associated fibula fractures underwent fibula fixation first with the help of distal fibula plates or one-third tubular plates to maintain their length. Then, the tibia was reduced and fixed based on the fracture pattern through an Antero medial approach, Antero lateral, or posterior medial approach. Distal tibia plates (anatomical antero lateral plates, medial plates, Ellis plates), lag screws, and K-wires were used for the fixation. Few patients with a high degree of comminution and impaction resulting in bone loss of the metaphyseal region required incorporating a tricortical iliac crest bone graft to maintain the length and alignment (Figure 2).

Skin condition was observed post-surgery for 2-3 days with foot end elevation and immobilization maintenance. Patients were discharged after getting the post-fixation radiographs. These patients were followed up after 10 days of surgical management for suture removal and then every four weeks for a minimum of six months with serial radiographs. The ankle ROM starts when the soft tissue is healed after two to four weeks. Weight-bearing is advised six to eight weeks post-surgery after observing the radiological signs of the union.

Patients are evaluated functionally using the AOFAS scoring system after a minimum follow-up of six months. This system comprises nine parameters across three categories, i.e., pain (40 points), function (50 points), and alignment (10 points), which sums up to a total of 100 points. A total score of 80-100 is considered to have an excellent functional outcome, scores ranging from 60-80 are good functional outcomes, scores between 40-60 are deemed satisfactory, and scores below 40 are considered poor functional outcomes [7,8].

Patients are also evaluated based on the ankle ROM using the reference values by Bone et al. [9], which are excellent range (dorsiflexion >10°, plantar flexion >30°), good range (dorsiflexion 5°-10° and plantar flexion >25°), moderate range (dorsiflexion 0°-5° and plantar flexion 20°), whereas poor range considered with dorsiflexion of 0° and plantar flexion < 20°.

The collected data was entered into an MS Excel sheet, and statistical analysis was done using the Statistical Package for Social Sciences software version 18. Descriptive statistics were employed to summarize the quantitative variables of demographic and clinical data. Standard deviation was calculated as a measure of variation. Qualitative variables were expressed as percentages with 95% CI. The level of significance (p-value) was set at p<0.05.

## Results

**Figure 3:**
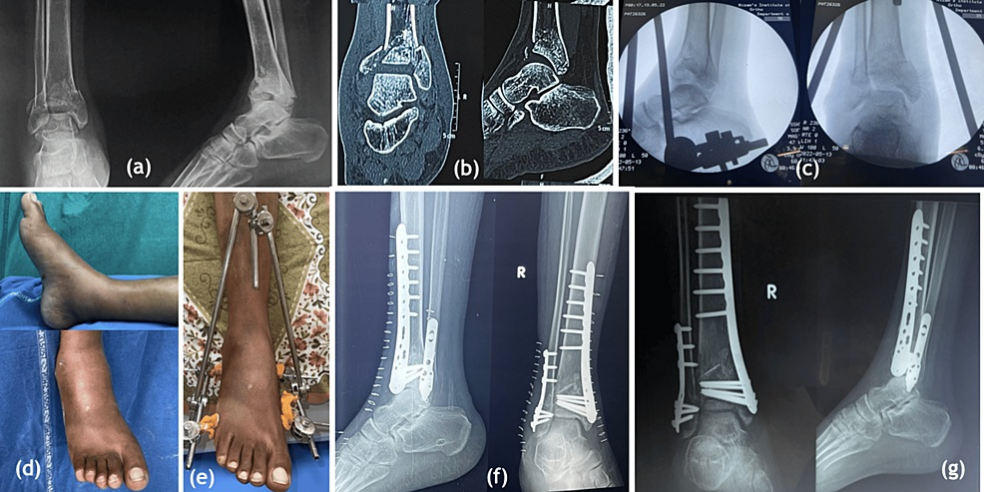
Staged management with initial spanning external fixator application followed by definitive fixation. (a) AP and lateral radiographs on presentation, (b) CT scan showing impaction of the articular surface on coronal and saggital sections, (c) AP and lateral radiographs post spanning external fixator application, (d) swelling on day of presentation, (e) appearance of wrinkles on day 3 of application of external fixator, (f) AP and lateral radiographs immediately after definitive fixation with medial distal tibial locking plate and distal fibula anatomical plate with tricortical bone grafting, (g) AP and lateral radiographs after 3 months of definitive fixation. AP: anteroposterior; CT: computer tomography

In our study of 20 patients with tibial pilon fractures includes, four patients with open fractures and 16 patients with closed fractures were observed, with a minimum follow-up of six months. None of the patients are lost to follow-up. The mean age in the present study was 38.85 ± 13.33 years. The majority were between 20 and 39 years old, and this type of fracture is more common in active young adults, predominantly males. The majority of the patients sustained the fracture due to road traffic accidents (10 patients (50%)). Eight patients sustained a fracture due to a fall from height (40%), and the remaining two patients suffered due to slips and falls. Coming to the mode of fixation, most subjects underwent internal fixation (12 patients), five underwent external fixation, and one underwent K wire fixation. One subject underwent a combination of internal and external fixation as single-stage surgery, and one subject underwent a staged protocol of spanning external fixator application followed by definitive internal fixation (Figure 3).

The mean time interval from injury to surgery was 8.75±5.72 days, ranging from a minimum of one day to a maximum of 24 days due to delayed presentation. One patient underwent staged protocol surgical intervention, and for the rest, all single surgery was done.

As per AOFAS scoring system, all nine parameters are evaluated, and after a minimum of six months of follow-up, 55% of the subjects had good outcomes (11 subjects), followed by excellent outcomes in six subjects and satisfactory outcomes in three subjects (Table 1). None of the subjects had poor functional outcomes.

**Table 1:** Outcome as per AOFAS scoring system AOFAS: American Orthopaedic Foot and Ankle Society

AOFAS scoring system	No of subjects
Excellent (80 -100)	6
Good (60-80)	11
Satisfactory (40 - 60)	3
Poor (<40)	0

As per Bone et al. [9] reference values, 45% of the subjects had excellent ROM (Dorsiflexion > 10° & Plantarflexion > 30°) (9 subjects), good total ROM (Dorsiflexion 5°-10° & Plantarflexion > 25°) in two subjects. Moderate total ROM in five subjects and poor total ROM at the ankle joint among four subjects, respectively, were documented during the final evaluation (Table 2).

**Table 2:** Evaluation based on Ankle ROM in subjects ROM - range of motion; DF - dorsiflexion; PF - plantar flexion

Total Ankle ROM	No of patients
Excellent (DF > 10^o^ & PF > 30^o)^	9
Good (DF 5^o^ - 10^o ^& PF > 25^o^ )	2
Moderate (DF 0^o^ - 5^o^& PF > 20^o^)	5
Poor (DF 0^o ^& PF < 20^o ^)	4

The mean time interval between injury and surgery among those with the excellent functional outcome (11±7.155 days) was longer than those with the good functional outcome (8.75 ± 5.201 days) and those with the fair outcome(5.67±4.163 days)

**Figure 4:**
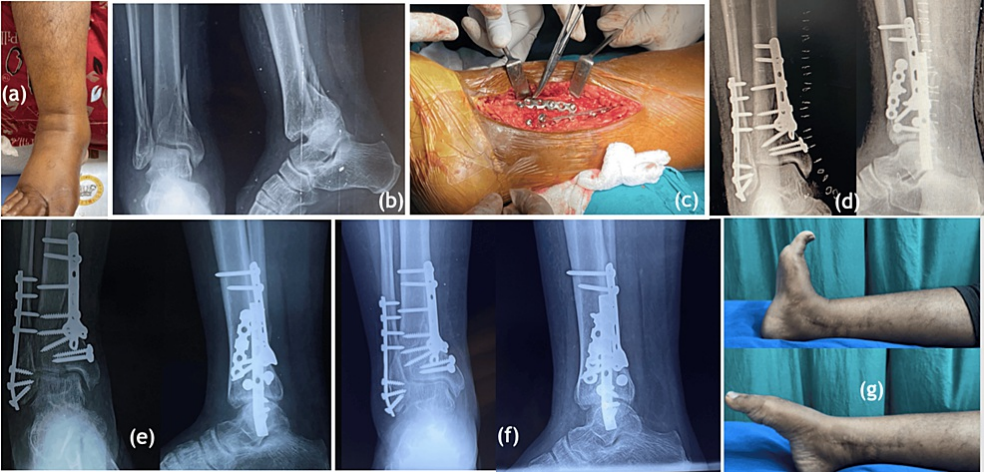
Patients with closed pilon fractures underwent internal fixation with excellent functional outcomes. (a) swollen ankle and foot, (b) AP and lateral radiographs on presentation, (c) internal fixation of the tibia through postero medial approach with Ellis plate and reconstruction plate, AP and lateral radiographs taken (d) on POD 2, (e) after 6 months of fixation, (f) after one year of fixation, (g) excellent ankle range of motion with dorsiflexion of 15° and plantar flexion of 35° AP: antero posterior; POD: post-operative day

Among those with excellent and good outcomes, the majority of the patients (14) had closed fractures (82%) and undergone internal fixation (60%) (11 subjects) when compared to the other modes of fixation (Figure 4).

80% of cases managed by external fixation show excellent and good functional outcomes combined, whereas 92% of cases managed by internal fixation show excellent and good functional outcomes combined (Table 3).

**Table 3:** Mode of fixation in subjects with the type of fracture, the resulting functional outcome, and complications K-wire: Kirschner wire; AOFAS: American Orthopaedic Foot and Ankle Society

Mode of fixation	No.of patients with Open/Closed fracture		Functional Outcome based on AOFAS score			No. of Patients with Complications
External	Open– 3	Closed- 2	Excellent– 1 (20%)	Good- 3 (60%)	Satisfactory- 1 (20%)	Varus malunion-1 Ankle stiffness-1
Internal	Open– 0	Closed- 12	Excellent – 3 (25%)	Good- 8 (66.66%)	Satisfactory- 1 (8.33%)	Ankle stiffness-1
External + Internal	Open– 0	Closed- 1	Excellent- None	Good- None	Satisfactory - 1 (50%)	Delayed wound healing and deep infection -1
Staged protocol	Open– 0	Closed- 1	Excellent – 1 (100%)	Good- None	Satisfactory- None	
K – wire	Open– 1	Closed- 0	Excellent - 1 (100%)	Good- None	Satisfactory- None	

The association of factors with the functional outcome among patients with tibial pilon fracture, such as age, mode of injury, type of fracture, and mode of fixation, is as follows. Among those with excellent outcomes, the majority belonged to the age group of 20 - 39 years (50%) than when compared to other age groups, the majority of the patients sustained a tibial pilon fracture due to a road traffic accident (50%) than when compared to other modes of injury i.e., either fall from height (43.75%) or slip and fall (6.25%), major subset of the patients had closed fractures than when compared to those with open fractures.

In patients with good outcomes, the majority had closed fractures in a similar fashion with satisfactory outcomes, too. Among those with satisfactory outcomes, all the patients had type III fractures, and none of them had type I or II, as per Reudi and Allgower (Table 4). Among those with excellent and good outcomes, the majority of the study subjects had undergone internal fixation (60%) than when compared to the other modes of fixation.

**Table 4:** Association of various factors with the functional outcome among patients with tibial pilon fractures FO: Functional Outcome

Factor	Excellent FO	Good FO	Fair FO	Total
Age vs Functional Outcome				
< 20 years	1 (16.7%)	0 (0%)	0 (0%)	1 (5%)
20 - 39 years	3 (50%)	6 (54.5%)	2 (66.7%)	11 (55%)
40 - 59 years	2 (33.3%)	3 (27.3%)	1 (33.3%)	6 (30%)
≥ 60 years	0 (0%)	2 (18.2%)	0 (0%)	2 (10%)
Mode of Injury vs Functional Outcome				
Road Traffic Accident	3 (50%)	6 (54.5%)	1 (33.3%)	10 (50%)
Fall from height	2 (33.3%)	4 (36.4%)	2 (66.7%)	8 (40%)
Slip and fall	1 (16.7%)	1 (9.1%)	0 (0%)	2 (10%)
Type of Fracture vs Functional Outcome				
Open	1 (16.7%)	2 (18.2%)	1 (33.3%)	4 (20%)
Closed	5 (81.3%)	9 (81.8%)	2 (66.7%)	16 (80%)
Type of fracture as per Reudi and Allgower of Fracture vs Functional Outcome				
Type I	1 (16.7%)	1 (9.1%)	0 (0%)	2 (10%)
Type II	1 (16.7%)	4 (36.4%)	0 (0%)	5 (25%)
Type III	4 (66.6%)	6 (54.5%)	3 (100%)	13 (65%)
Mode of Fixation vs Functional Outcome				
External	1 (16.7%)	3 (27.3%)	1 (33.3%)	5 (25%)
Internal	3 (50%)	8 (72.7%)	1 (33.3%)	12 (60%)
External + Internal	1 (16.7%)	0 (0%)	1 (33.3%)	2 (10%)
K wire	1 (16.7%)	0 (0%)	0 (0%)	1 (5%)
Total	6 (100%)	11 (100%)	3 (100%)	20 (100%)

**Figure 5:**
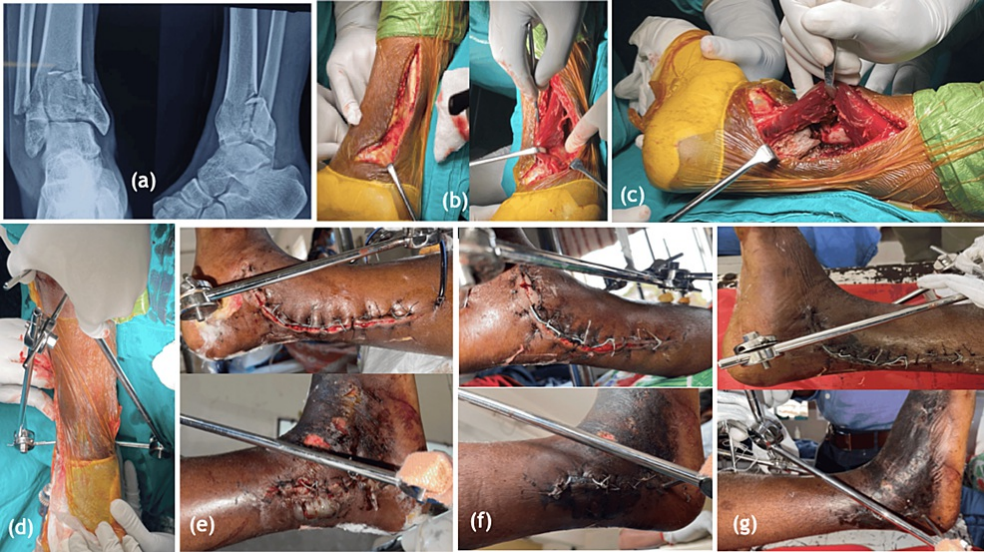
Complication of delayed wound healing and development of blebs. (a) AP and lateral radiographs on presentation, (b) posteromedial approach, (c) elevation of FHL muscle to reduce fracture and plate fixation, (d) external fixation for additional stability, (e) development of blisters and swelling on POD 2, (f) Skin condition on POD 7, no blisters, (g) Skin condition on POD 16 AP: antero posterior; POD: postoperative day

Complications of varus malunion in one and ankle stiffness were found in two patients; one was managed by external fixation and another by internal. One patient had delayed wound healing with post-operative blebs and developed a deep infection after undergoing a combined fixation of internal and external for additional stability and soft tissue healing (Figure 5). Further treatment is by implant removal, debridement, and external fixation.

## Discussion

Pilon fractures are known for their dilemma in management options. The surgeon has to manage and maintain a balance between the bony and soft tissue injuries. When choosing a management option for pilon fractures, it is essential to consider not just fracture stability but also associated soft tissue injury, which often leads to additional complications such as wound dehiscence and infection [3,10]. 

Our study shows that during the study period and at the place where the study was conducted, tibial pilon fractures were more prevalent in men (95%), which was slightly more compared with what has been reported in the literature in a study by Collinge et al. male prevalence is 67% while the female is 33% [11]. Road traffic accidents were the main mode of injury (50%) in our study, which is slightly low compared to the study by Moura Junior AF and Machado in 2018 [12], where motorcycle accidents caused 81% of pilon fractures, which correlates with the current epidemiological data [13,14] followed by fall from height (40%) and slip and fall (10%). The mean age of the 20 patients studied was 38.85±13.335 years, with the youngest being 19 years and the oldest being 62 years; when compared with studies by Baris A et al. [15] and Kishore et al. [16], the mean age of patients was 45.3 years and 47 years, which was similar to our study. Inferring that most victims belong to the working-age population with a long life expectancy and that these fractures lead to a reduction in their earnings. Based on the study, young males with road traffic accidents are more prone to fractures.

The mean time interval between the injury and surgical intervention was 8.75±5.72 days, ranging from a minimum of one day to a maximum of 24 days due to delayed presentation of a patient, which is consistent with the findings of other authors [17-20]. This time interval is essential to restore the surrounding soft tissue, significantly reducing associated complications.

There are four subjects with open fractures, where three are managed with external fixation, one managed with K-wire fixation, and 16 with closed fractures. Two subjects are managed with an external fixator, one with internal and augmented with external fixation and the rest by internal fixation. Two subjects were managed by an external fixator even though it's a closed fracture, one due to delayed presentation with multiple blebs and considering his (CKD) chronic kidney disease condition requiring alternate day hemodialysis and the other because of associated compound femur fracture. For one subject with a closed fracture, internal fixation was done and augmented by an external fixator for additional stability and soft tissue healing. The remaining 12 subjects were managed by internal fixation, of which eight were managed by minimally invasive plate osteosynthesis (MIPO) technique medial plating, and the remaining four were managed with open reduction internal fixation.

In the present study, fibula fixation was done depending on the fracture pattern. In cases with bone loss, bone grafting with the iliac crest is done. Non-weight bearing was advised for these cases till signs of fracture union were seen. In clinical terms, the union is characterized by a pain-free fracture site on bearing weight. Radiologically, a fracture is deemed united if there is no discontinuity in three out of four cortices in two orthogonal radiographic views. Failure to achieve union by the end of six months post-surgery is labeled delayed union, while non-union is designated if the union is not achieved by the ninth month [21]. The mean follow-up time was nine months, ranging from 6 to 20 months in the study.

As per the AOFAS scoring system, the majority (10 patients) had occasional mild pain under the pain category. None of the subjects had complained of severe pain. Under the category of function, the majority had no activity limitation and no support requirement (10 patients), were able to walk for a maximum distance of >6 blocks (15 patients), had no difficulty walking on any surface (14 patients), had no or slight gait abnormality (16 patients), had normal or mild restricted sagittal motion (flexion+extension) (12 patients), had normal hind foot motion (inversion+eversion) (10 patients) and all had stable ankle-hind foot (20 patients). Under the category of alignment, the majority had good alignment with plantigrade foot (17 patients). The majority had good outcomes (11 patients), followed by excellent outcomes (6 patients) and satisfactory outcomes in three patients. None had poor functional outcomes.

Our study's mean AOFAS score is 85.60±11.68, which correlates with Hong et al. [18], whose study is on both extra and intraarticular distal tibia fractures and obtained a mean AOFAS score of 87.3 points. In a study published in 2022, 86.75% excellent to good outcomes by the AOFAS scoring system has been comparable to the present study [21].

In our study, the mean fracture healing time was 3.55±1.538 months, which was slightly less than that by Bone et al. [9] in their study (4.5 months). In patients with excellent outcomes, the time of union is 3.00±1.09 months, which is lesser than those with good and fair outcomes, i.e., 3.55±1.83 and 3.67±1.15, respectively.

In our study, excellent and good outcomes were seen in 81% of patients in the younger population aged 20-39 years. In patients with open fractures, excellent and good outcomes were seen in 75%. In closed fractures, excellent and good functional outcomes are seen in 87.5%. In patients under type I and type II categories of Reudi and Allgower classification, no satisfactory and poor functional outcomes. In type I, excellent outcomes are seen in one patient and good in one patient. Excellent outcomes were seen in 20% of subjects of Type II Reudi and Allgower, and good outcomes in 80% of the subjects of Type II Reudi and Allgower. In the Type III category, 30.7% have an excellent functional outcome, 46% have a good functional outcome, 23% have a satisfactory functional outcome, and none have a poor outcome. This shows that severely comminuted fracture patterns due to high energy impact are associated with decreased functional outcomes.

There were no intraoperative complications encountered. None of the subjects had wound dehiscence or implant exposure. While pus discharging sinus was found in a subject who sustained type III Reudi and Allgower fracture. He presented with pre-operative blebs and had delayed wound healing with post-operative blebs.

In two subjects 10° malunion found in sagittal plane with posterior angulation of the tibia. One subject has 5° of varus malalignment, which was an open injury managed by an external fixator. It was insignificant clinically, as the patient didn't report any difficulty. Ankle stiffness developed in two subjects, one treated with external fixation and the other with the MIPO technique. This is attributed to the patient's compliance with the recommended physiotherapy schedule. Additionally, two subjects experienced ankle edema, which was managed by compression elastic bandage and medication. No other complications like reflex sympathetic dystrophy (RSD) or early arthritis were found in the study.

Limitations

This is a small study with a sample size of 20 patients. It is a short-term study with a minimum follow-up period of 6 months. Long-term complications such as arthritis couldn't be assessed. The functional outcome has been evaluated, but the radiological accuracy of reduction hasn't been evaluated. The effect of fracture management on the patient's quality of life isn't evaluated.

## Conclusions

Tibial pilon fractures are complex injuries. Management should be patient-specific as limited soft tissue coverage, poor vascularity in the region, and the subcutaneous nature of the bone continues to be detrimental. The timing of surgery should be optimized to allow the soft tissues to stabilize. Tibial pilon fractures are more common in young adults as a result of road traffic accidents, the most common type being the closed and type III, as per Reudi and Allgower's classification. A positive wrinkle sign should be important to proceed with definitive management. Definitive internal fixation after soft tissue healing has shown excellent and good functional outcomes (per AOFAS score) with better ankle ROM and without complications compared to external fixation, where ankle stiffness and delayed union are encountered.
